# Polarized optical contrast spectroscopy of in plane anisotropic van der Waals materials

**DOI:** 10.1038/s41598-025-96894-8

**Published:** 2025-05-02

**Authors:** Ernst Knöckl, Alexandre Bernard, Alexander Holleitner, Christoph Kastl

**Affiliations:** 1 Walter Schottky Institute and Physics Department, Technical University of Munich, 85748 Garching, Germany; 2 Munich Center For Quantum Science and Technology (MCQST), Schellingstr. 4, 80799 München, Germany

**Keywords:** Microscopy, van der Waals materials, Birefringence, Optical anisotropy, Optical spectroscopy, Materials science, Surfaces, interfaces and thin films, Micro-optics

## Abstract

Polarized optical contrast spectroscopy is a simple and non-destructive approach to characterize the crystalline anisotropy and orientation of two-dimensional materials. Here, we develop a 3D-printed motorized polarization module, which is compatible with typical microscope platforms and enables to perform broadband polarization-resolved reflectance spectroscopy. As proof of principle, we investigate the in-plane birefringence of exfoliated $\hbox {MoO}_3$  thin films and few-layer $\hbox {WTe}_2$  crystals. We compare the measured spectra to a model based on a transfer matrix formalism. Compared to other polarization sensitive approaches, such as Raman or second harmonic generation spectroscopy, optical contrast measurements require orders of magnitude less excitation power densities, which is particularly advantageous to avoid degradation of delicate van der Waals layers.

## Introduction

Two-dimensional (2D) layered materials are studied both for their intriguing physics and for their potential applications in (opto)electronics and quantum technologies^1,2^. Taking advantage of the weak van der Waals (vdW) force between the atomically thin layers, they can be exfoliated and combined with other 2D materials into vdW heterostructures^3^, with a plethora of emergent optical and electronic properties. Very often, such emergent behavior originates from the breaking of electronic symmetries. In this context, recent studies highlighted the possibility to control the interfacial symmetries by combining a low-symmetry layer, in optical terms an anisotropic or birefringent material, with a high-symmetry layer, in optical terms an isotropic material^4–6^.

To properly exploit such interfacial symmetry breakings, it is crucial to determine the symmetry axes of the individual layers and their respective alignments. A common approach is to initially align the layers in the heterostructure based on the shape and edge orientation of the exfoliated crystals as a rough estimate. Subsequently, one can use polarization sensitive spectroscopy methods for refined measurements of the crystal alignment. Amongst suitable methods are polarized second harmonic generation and Raman spectroscopy^7–10^. However, the underlying (non-linear) microscopic processes tend to be inefficient, often requiring relatively high power densities of up to $P = 10\hbox {m}\, \hbox {W} \upmu \hbox {m}^{-2} = 10\hbox {k}\, \hbox {W}\hbox {cm}^{-2}$, the equivalent of $10^5$ suns at AM 1.5 G conditions, to establish the polarization dependent signal with sufficient confidence. Such high optical powers can easily deteriorate microscopic samples, especially for excitation above their fundamental gap, where absorption becomes relevant. With a $10^{6}$ less powerful light source, polarized optical contrast imaging is a common and effective technique to study the crystalline orientation of inorganic^11^ and organic^12^ thin films.

Here, we demonstrate a simple and cheap, yet effective, approach for quantitative polarized optical contrast spectroscopy of vdW materials and their heterostructures that integrates seamlessly with typical microscope platforms. We exploit the built-in white light illumination of a commercial confocal Raman microscope for scanning reflectance spectroscopy with micrometer spatial resolution, similar to Ref.^13^. By introducing a 3D-printed, motorized polarization module in the illumination pathway, we enable non-destructive polarized contrast spectroscopy to determine the crystalline orientation of sensitive, atomically thin vdW materials. As proof of principle, we first study $\hbox {MoO}_3$  as a typical optically transparent and birefringent vdW insulator, and we compare the polarized contrast spectroscopy to polarized Raman spectroscopy. As an example of a more delicate material, we then investigate semimetallic, few-layer $\hbox {WTe}_2$, where Raman spectroscopy often turns out to be destructive due to the non-negligible absorption of $\hbox {WTe}_2$  in the visible range. For both of the above material systems, we compare the measured reflectance spectra to a 4×4 transfer matrix model^14^, which not only provides thickness estimation with precision comparable to atomic force microscopy, consistent with other specific implementations of polarized contrast spectroscopy^13,14^, but is also important for reliable assignment of the crystal axes.

**Fig. 1:**
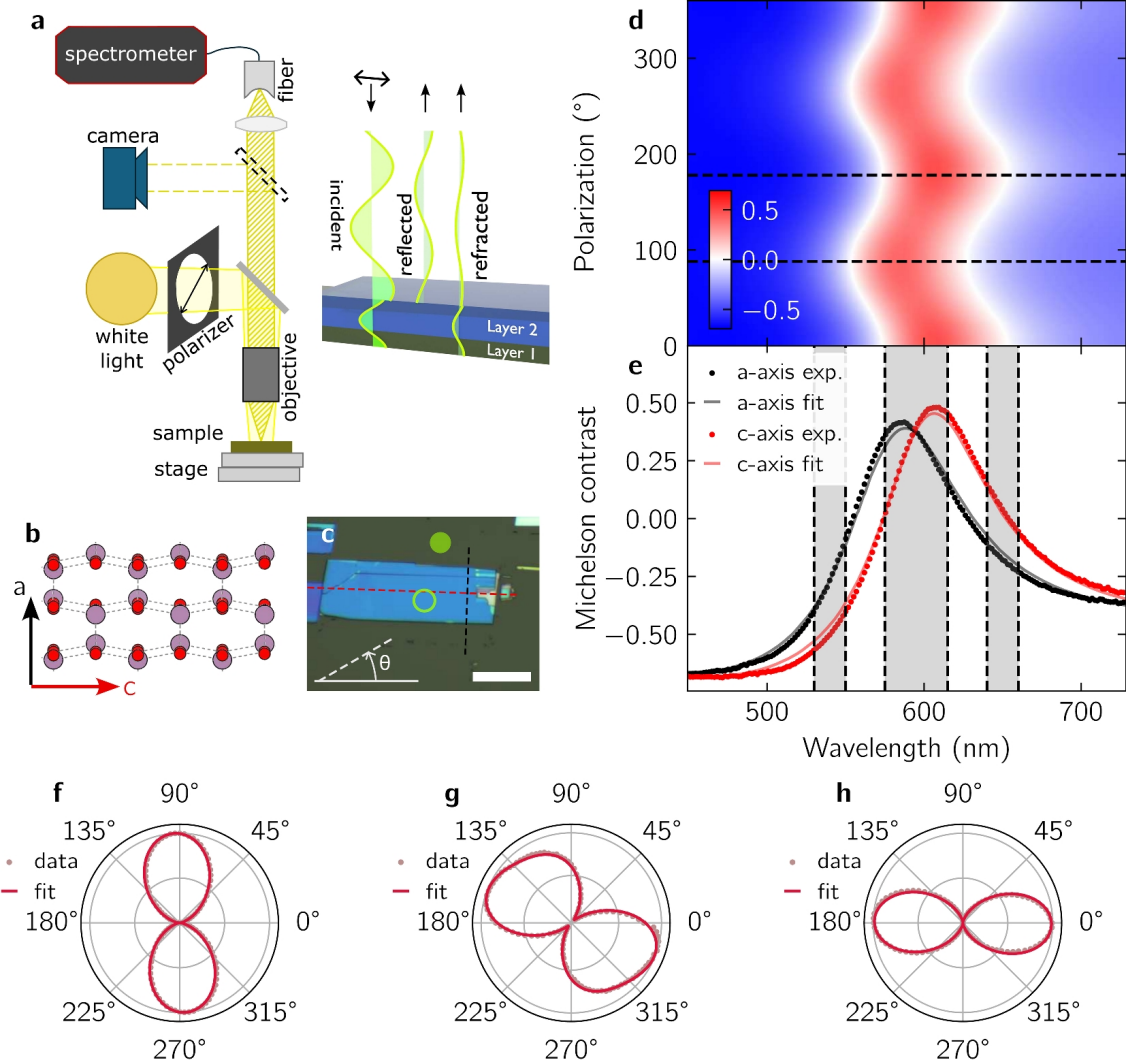
(**a**) Polarized white light illuminates the sample in a wide-field configuration. Light from the diffraction-limited focus spot is collected by a single-mode fiber and analyzed in a spectrometer. In a multi-layer structure, thin-film interference determines the reflectance contrast. (**b**) Crystal structure of $\alpha -$ $\hbox {MoO}_3$ viewed perpendicular to the vdW planes. Red (purple) disks represent oxygen (molybdenum) atoms. The crystallographic a-axis and c-axis are distinct leading to birefringence. (**c**) Optical image of a $\hbox {MoO}_3$  flake on a Si/$\hbox {SiO}_2$  substrate with the reference spot ($R_\text {off}$, full disk) and the sample spot ($R_\text {on}$, empty circle) used for defining the Michelson contrast. The red (blue) dashed line aligns with a-axis (c-axis). The polarization angle $\theta$ is defined as indicated. Scale bar, 20 $\upmu \hbox {m}$. (**d**) Contrast spectra for various polarization orientations $\theta$. (**e**) Michelson contrast spectra measured at $88^\circ$ (black dots) and $178^\circ$ (red dots) as indicated by the dashed lines in (**d**). The solid lines are fits with the transfer matrix model. (**f**, **g**, **h**) Normalized polar plots of the contrast integrated over different wavelength regions, represented by the three grey areas in (**e**). The solid lines are fits with sinusoidal functions.

## Results

Orthorhombic $\hbox {MoO}_3$, or $\alpha$-$\hbox {MoO}_3$, has found recent interest due to its strongly anisotropic optical properties, enabling, for example, hyperbolic polaritons^15^. Furthermore, the anisotropic reflectance of $\hbox {MoO}_3$ can be exploited for polarization sensitive reflectors and color filters^16^. Several studies already analysed the (anisotropic) optical properties of $\hbox {MoO}_3$^16–18^. For example, Puebla et al. combined unpolarized optical contrast with a simple Fresnel analysis to determine the thickness of thin $\alpha$-$\hbox {MoO}_3$ on Si/$\hbox {SiO}_2$ substrates^17^. In a follow-up study, this approach was extended to explicitly extract the angle dependence of the in-plane refractive index^18^. Figure 1a sketches the principle of our implementation of reflectance contrast spectroscopy, which unifies several aspects of the aforementioned works on $\hbox {MoO}_3$ and which will serve as the basis for discussing general requirements for reliable determination of the crystal axes orientation of birefringent vdW dielectrics on multi-layer substrates or embedded into heterostructures. White-light from an LED is sent through a custom-made module (Supporting Figure S1), which inserts a rotatable linear polarizer into the beam path. The polarized light illuminates the sample at approximately normal incidence. The output power density on the sample is on the order of ${2}{\hbox {n}\, \hbox {W} \upmu \hbox {m}^{-2}}$, which is orders of magnitude lower than in typical Raman or second Harmonic generation experiments^8,19^. For birefringent thin films, such as the $\hbox {WTe}_2$  and $\hbox {MoO}_3$  studied here, the electric field components along the two optical axes experience different refractive indices, which includes both *n* (propagation) and *k* (absorption). Moreover, both refractive indices depend on the wavelength (Supporting Figure S2). In a heterostructure or on a substrate, the birefringence results in modified interference conditions along the crystal axes and, therefore, different reflected intensities. In our case, the reflected light is collimated by an objective lens ($10\times$ with numerical aperture $\textrm{NA}=0.25$ for the $\hbox {MoO}_3$, $50\times$ with $\textrm{NA}=0.5$ for the $\hbox {WTe}_2$) and collected by a single-mode optical fiber. Due to the fiber-based detection, only light from the diffraction limited focal spot is analyzed in the spectrometer. The detection is unpolarized. To achieve spatial imaging with sub-micron resolution, a motorized xy-stage can move the sample relative to the focus spot.

To define a differential reflectance contrast, two reflectance spectra are measured for each polarization orientation $\theta$. One spectrum is acquired on a sample spot including the birefringent material ($R_{\textrm{on}}(\lambda , \theta )$), and another one is acquired on a reference spot within the same heterostructure but excluding the birefringent material ($R_{\textrm{off}}(\lambda , \theta )$). Then, the corresponding Michelson contrast is computed as follows:

1 $$\begin{aligned} C(\lambda , \theta ) = \frac{R_{\textrm{off}}(\lambda , \theta ) - R_{\textrm{on}}(\lambda , \theta )}{R_{\textrm{off}}(\lambda , \theta ) + R_{\textrm{on}}(\lambda , \theta )}, \end{aligned}$$

where $\lambda$ is the wavelength of the light and $\theta$ is the angle of the polarization in the sample plane, as defined in Fig. 1c. Generally, $C(\lambda , \theta )$ can range from $-1$ to $+1$, where $-1$ corresponds to no reflected signal from the reference spot ($R_{\textrm{off}}=0$), and $+1$ corresponds to no reflected signal from the sample spot ($R_{\textrm{on}}=0$). The measurement on the reference spot eliminates the polarization and wavelength dependencies of the optics and the white light illumination, which would otherwise hamper quantitative measurements.

As a first example, we study the anisotropic contrast of $\alpha -$ $\hbox {MoO}_3$, see Fig. 1b for its crystal structure. Thin layers of $\hbox {MoO}_3$  are exfoliated onto Si/$\hbox {SiO}_2$  (oxide thickness 70 nm). Figure 1c shows an optical image of the structure. The empty and full disks indicate the position where the sample and reference spectrum are measured, respectively. The polarization angle $\theta$ is defined as indicated. Figure 1d shows the polarization-dependent Michelson contrast of a 146 nm thick $\hbox {MoO}_3$. In this representation, the most prominent effect of rotating the polarization is to red- or blue-shift the contrast spectrum. We find a clear angular dependence with two distinct axes at ${88}^{\circ }$ and ${178}^{\circ }$, which we tentatively assign to the main optical axes of $\hbox {MoO}_3$  based on the rectangular shape of the flakes (Fig. 1c).

To model the contrast spectra, we use a 4×4 transfer matrix formalism, which accounts for the anisotropic refractive indices in the layers of the heterostructure, as discussed for example in Refs.^14,20^. Figure 1e compares the experimentally measured spectra (dots) and the fitted model (solid lines). The refractive indices of $\hbox {MoO}_3$  are taken from Ref.^21^ (see also Supporting Figure S2). For Si and $\hbox {SiO}_2$, we use the values provided in Refs.^22^ and^23^. As free model parameters, we use the thickness of the individual layers. The model confirms that ${88}^{\circ }$ and ${178}^{\circ }$ correspond to the a- and c-axis. The fit provides an estimate of ${\hbox {(}142\pm 1\hbox {)}}\,{\hbox {nm}}$ for the thickness of $\hbox {MoO}_3$, matching reasonably well the ${\hbox {(}146\pm 3\hbox {)}}\,{\hbox {nm}}$ measured by atomic force microscopy, as well as ${\hbox {(}73\pm 1\hbox {)}}\,{\hbox {nm}}$ for the $\hbox {SiO}_2$  layer, close to the nominal value of ${\hbox {(}70\pm 3\hbox {)}}\,{\hbox {nm}}$. Small deviations of model and data may stem from uncertainties in the refractive indices used to simulate the light propagation through the stack. The impact of finite numerical aperture, or equivalently the impact of illumination under oblique angle of incidence^14^, can be minimized by selecting a small aperture stop for the illumination. Effectively, this realizes an almost ideal wide-field illumination with perpendicular angle of incidence, even for a $100\times$ objective lens with $\text {NA} = 0.9$ (Supporting Figure S4).

To identify the crystal orientation with high fidelity and precision, we discuss the angular dependence of the contrast integrated over different wavelength regions, represented by the three grey-shaded areas in Fig. 1e. The corresponding polar plots are depicted in Fig. 1f–h, where all amplitudes are normalized. At first order, the variation of the contrast with polarization angle can be fitted with a ${180}^{\circ }$-periodic sinusoidal function. But generally, the full response integrated over a fixed wavelength interval is not a simple scalar product between the electric field and the crystal axis vectors (e.g. the blue or red shift of a non-linear curve here). For this reason, the experimental data in the polar plots are best fitted with a ${180}^{\circ }$-periodic sinusoidal function plus a smaller second harmonic (solid lines). Figure 1f,h yield a very clear anisotropic response where the main axis of the corresponding polar plot is oriented along the a-axis and c-axis, respectively, in line with previous studies^18^. By contrast, Fig. 1g exhibits a deformed polar pattern, where the crystal axis cannot be directly inferred. This is due to a poor choice of spectral region of interest located between the contrast peaks of the a- and c-axis. Combined with the transfer matrix method, this result demonstrates the ability of polarized optical contrast spectroscopy to identify the crystal axis of a birefringent material at a specific wavelength. However, care must be taken to select a wavelength window which is well separated from the main contrast peak for all polarization angles. For transparent birefringent materials with negligible absorption, such as $\hbox {MoO}_3$, this can be understood by looking again at Fig. 1d. As the polarization angle rotates in-plane, the contrast spectrum shifts in wavelength, yet its shape is barely affected. The integration windows in Fig. 1f,h are selected to maximize the derivative of the contrast spectrum with respect to wavelength, which, consequently, yields maximum sensitivity to changes in the polarization angle. We note that as the film thickness changes, the interference conditions and consequently the polarized reflectance spectrum are modified substantially. However, taking into account the above consideration about selecting a proper wavelength window, crystal axis read-off by polarized optical contrast remains precise (c.f. Supporting Figure S5 for contrast measurements on a $\hbox {MoO}_3$ film featuring thickness steps of 12 nm, 40 nm and 111 nm).

**Fig. 2:**
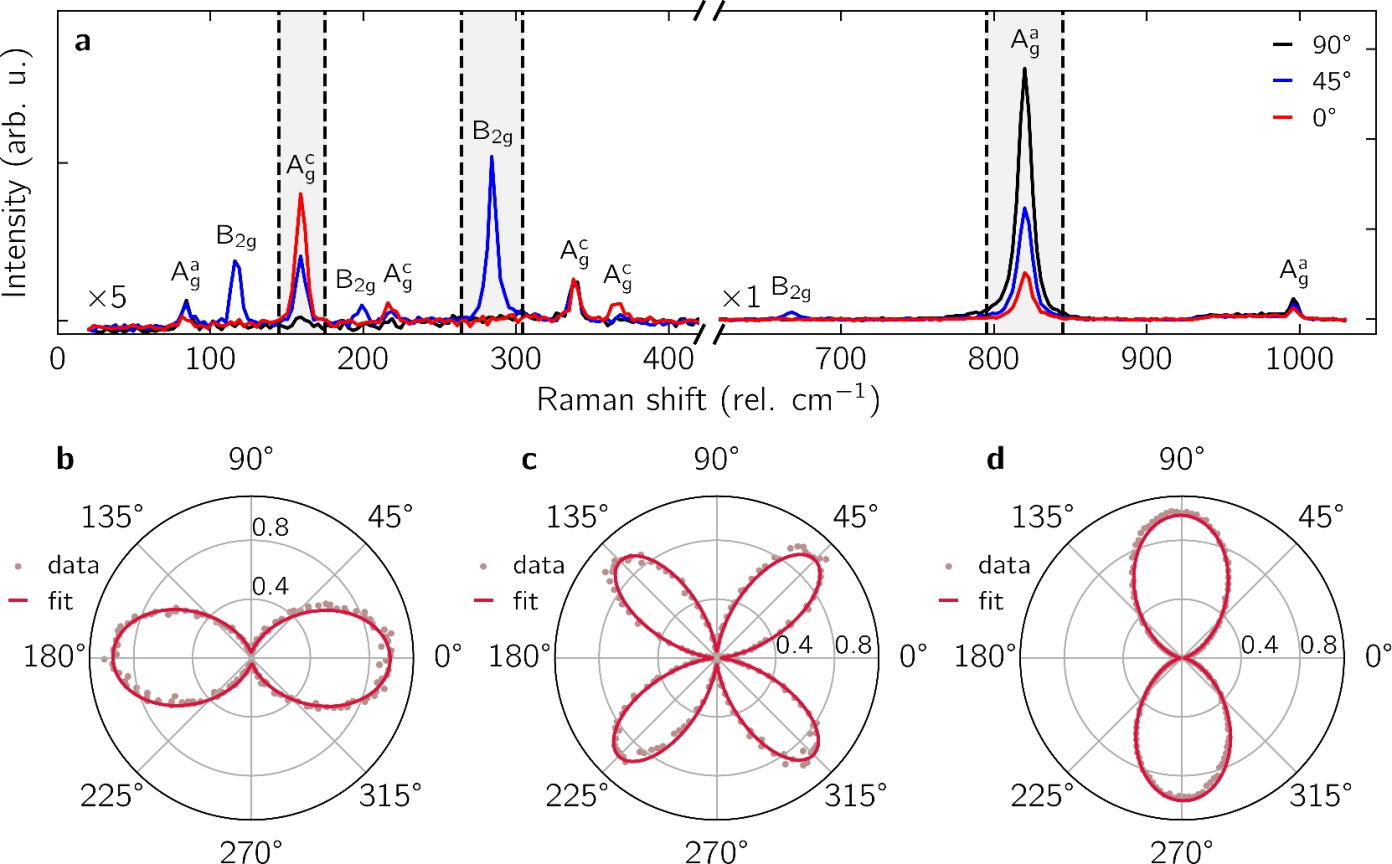
(**a**) Angle-resolved polarized Raman spectrum of the $\hbox {MoO}_3$  flake for polarization angle ${0}^{\circ }$ (red line), ${45}^{\circ }$ (blue line), and ${90}^{\circ }$ (black line). Peaks are labeled with the corresponding phonon mode following Ref.^24^, and with the measured peak center. The Raman modes below $400\, {\hbox {cm}}^{-1}$ were rescaled by a factor 5. (**b**, **c**, **d**) Polar plots of the integrated, normalized peak intensity for modes ${\hbox {A}}_{g}^{c}$, ${\hbox {B}}_{2g}$, and ${\hbox {A}}_{g}^{a}$ as highlighted by the grey shaded areas in (**a**).

To compare with other methods, we performed angle-resolved, polarized Raman spectroscopy on the same $\hbox {MoO}_3$  flake (Fig. 2). We used a 532nm excitation with $P = 5\hbox {m}\, \hbox {W}$ in confocal configuration with a $10\times$ objective with numerical aperture $\textrm{NA}= {0.25}$, yielding a power density $P \simeq {5.6}{\hbox {m}\, \hbox {W}}\upmu {\hbox {m}}^{-2}$ and a beam spot of $\sim {1}\,{\upmu \hbox {m}}$. The Raman measurement is done at ambient conditions. Figure 2a displays the co-polarized Raman spectrum obtained at ${0}^{\circ }$, ${45}^{\circ }$ and ${90}^{\circ }$ polarization angles after integrating for 5 s. To enhance the visibility of the Raman modes below $400\, {\hbox {cm}}^{-1}$, the corresponding spectra are scaled by a factor 5. The spectrum is consistent with previous reports^18,24,25^. The full data set for co- and cross-polarized detection and the peak positions are reported in Supporting Figure S3.

Figure 2b–d exemplarily depict polar plots of the integrated peak intensity for the $\hbox {A}_g^c$ ($115\, {\hbox {cm}}^{-1}$), $\hbox {B}_{2g}$ ($283\, {\hbox {cm}}^{-1}$), and $\hbox {A}_g^a$ ($818\, {\hbox {cm}}^{-1}$) mode, respectively. For the polar plots, all amplitudes are normalized, and the solid lines are fits of the experimental data with sinusoidal functions. In agreement with earlier studies, the Raman intensity of the $\hbox {A}_g^c$ and $\hbox {A}_g^a$ modes exhibit a ${180}^{\circ }$ periodic pattern maximized along the c-axis and a-axis, respectively. By contrast the $\hbox {B}_{2g}$ signal has a ${90}^{\circ }$ periodicity, where the intensity is maximized at ${45}^{\circ }$ away from the crystal axes. Therefore, in the case of $\hbox {MoO}_3$, it is equivalently possible to deduce the crystal orientation from the polarization dependence of the Raman spectrum. In general, higher laser powers and longer integration times are required to reach the same signal to noise ratio as with polarized optical contrast.

**Fig. 3:**
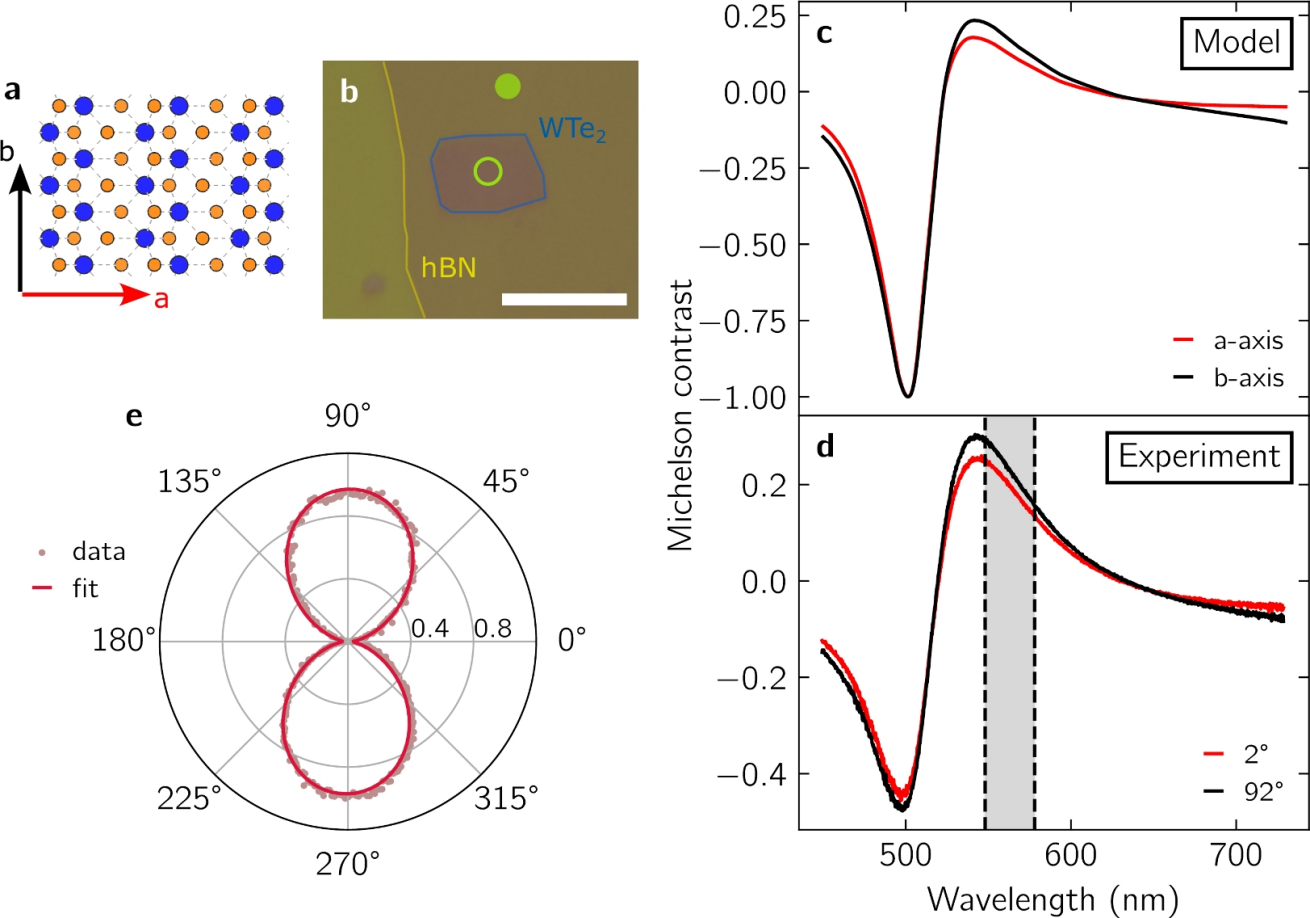
(**a**) Crystal structure of $\hbox {WTe}_2$  viewed perpendicular to the vdW plane. Blue (orange) disks represent tungsten (tellurium) atoms. (**b**) Optical image of a $\hbox {WTe}_2$  flake on a suspended silicon-rich nitride (SiRN) membrane and capped with hexagonal boron nitride (hBN) with the reference spot ($R_\text {off}$, full disk) and the sample spot ($R_\text {on}$, empty circle). Scale bar, 10 $\upmu \hbox {m}$. (**c**) Michelson contrast spectra calculated by the transfer matrix model for a-axis (red line) and b-axis (black line), with SiRN thickness of 220 nm, $\hbox {WTe}_2$  thickness of 3.9 nm, and hBN thickness of 4 nm. (**d**) Michelson contrast spectra measured at ${2}^{\circ }$ (red line) and ${92}^{\circ }$ (black line). (**e**) Normalized polar plot of the contrast integrated over the 30 nm interval represented by the grey area in (**c**). The solid line is a fit with sinusoidal functions.

Next, we discuss $\hbox {WTe}_2$  as an example of an anisotropic semimetal^26^. $\hbox {WTe}_2$  draws attention owing to its topological properties. Monolayer $\hbox {WTe}_2$  was shown to be a quantum spin-Hall insulator with topologically protected helical edge states^27,28^. Bulk $\hbox {WTe}_2$  shows theoretical and experimental evidence of being a so called type-II Weyl semimetal^29,30^ coexisting with higher-order-topology-induced helical hinge states^31,32^. Few-layer $\hbox {WTe}_2$  constitutes an intermediate case, where the only true symmetry is a single in-plane mirror axis. In the latter case, the large anisotropy of the lattice enhances the so called Berry curvature dipole, which manifests as crystal axis dependent non-linear anomalous Hall effect^33–36^.

A top view of its crystal structure is depicted in Fig. 3a, highlighting the two distinct crystal axes. Figure 3b shows an optical image of a heterostructure, where few-layer $\hbox {WTe}_2$  was transferred onto a suspended silicon-rich nitride (SiRN) membrane and subsequently encapsulated in hBN. This particular example highlights that the shape of the exfoliated $\hbox {WTe}_2$  crystal does not necessarily allow to infer the crystal axes, and that additional characterization methods are needed. Although polarized Raman spectroscopy or second harmonic generation are in principle possible^19,37^, the limited stability of few-layer $\hbox {WTe}_2$  under ambient conditions and laser irradiation^38^ necessitates relatively low optical powers (on the order of 100 $\upmu \hbox {W}$) and consequently long exposure times (on the order of 100 s)^19,37^ to establish the polarization dependence with high fidelity. For example, for our experimental configuration and samples, Raman spectroscopy at laser powers $P \simeq {1}{\hbox {m}\, \hbox {W}}$ led to visible damage in the optical images. By contrast, the reflectance contrast measurements are completely non-destructive and highly reliable, even for non-encapsulated films. Figure 3c,d depict the modelled and measured contrast spectra, respectively. The model uses the refractive indices of Refs.^39,40^ for hBN and $\hbox {WTe}_2$, respectively. For the specific SiRN substrates, the refractive index and thickness was independently measured via reflectometry. Importantly, we take into account the finite absorbance of $\hbox {WTe}_2$  in the modelling process (Supporting Figure S2). The model reproduces qualitatively the experimental curves with layer thicknesses of ${d}_{\text {hBN}} = 4 {\hbox {nm}}$, $d_{{\text {WTe}}_{2}} = {3.9}{\hbox {nm}}$ and $d_{\text {SiRN}} = {220}\,{\hbox {nm}}$. Compared to the simple example of $\hbox {MoO}_3$  on Si/$\hbox {SiO}_2$, the polarization angle and crystal axis have a more subtle effect on the spectra. The main effect of changing the polarization angle is not to shift the spectrum in wavelength, but to slightly rescale the relative amplitudes. For thin $\hbox {WTe}_2$  on a thick substrate, the principal shape of the contrast spectrum is dictated by the optical path in the substrate. For example the pronounced contrast minimum near 500 nm, which drops to $-1$ in the model and $-0.45$ in the measurement, corresponds to destructive interference at the surface of the reference spot. In a simplified picture, this occurs when the wavelength equals the optical length through the heterostructure and back. The latter value is only barely impacted by the birefringence introduced by the thin $\hbox {WTe}_2$  layer. Consistently, the contrast spectra along the a-axis and the b-axis are qualitatively very similar, and both refractive index and absorption need to be included in the model for the reliable assignment of the optical axes. To assign the crystal axes, we again integrate the contrast spectrum (Fig. 3e) over a suitable wavelength window (see grey-shaded area in Fig. 3d) as function of polarization angle. Figure 3e shows the obtained polar plot. The solid line is a fit of the data with sinusoidal functions. We find that the polarization at ${2}^{\circ }$ and ${92}^{\circ }$ orientations correspond to the a- and b-axis of the crystal, respectively. In contrast to the case of $\hbox {MoO}_3$, where the angular dependence at contrast maximum is ambiguous, selecting an integration window close to the contrast maximum yields good results for $\hbox {WTe}_2$. This can be understood by the fact that the polarization angle mainly scales the contrast amplitude, as discussed above.

## Conclusions

In summary, our study highlights that polarized optical contrast spectroscopy is an effective and non-destructive technique for characterizing the anisotropy of 2D and quasi-2D materials in vdW heterostructures both for ideal birefringent dielectrics (e.g. $\hbox {MoO}_3$), where absorption is negligible, and few-layered anisotropic semimetals (e.g. $\hbox {WTe}_2$), where a finite absorption needs to be considered. We confirm that the measured spectra can be modelled sufficiently well by the commonly used transfer matrix formalism, which requires only knowledge of the refractive index of the individual layers. While determination of the latter is typically straightforward for thick (bulk) van der Waals layers, the optical properties are often substantially modified in atomically thin layers and heterostructures thereof due to quantum confinement and hybridization effects^41^. In this context, spectroscopic imaging ellipsometry is a powerful tool to extract quantitative refractive index information of 2D materials and their heterostructures^42^. While many of the prototypical excitonic van der Waals materials, such as MoS_2_, WS_2_, MoSe_2_ or WSe_2_, do not exhibit birefringence by their crystal symmetry, our method can be relevant for strained materials or materials with a large anisotropy of the exciton transitions, such as the magnetic van der Waals semiconductor CrSBr^43^ or the hybrid layered material AgSePh^44^.

As an important aspect of the present work, we demonstrate a simple and cost-effective implementation of polarized contrast spectroscopy into standard optical microscope platforms based on a 3D-printed optics module. Our specific implementation cannot only be used to identify the alignment of the optical axes of a birefringent vdW layer in a heterostructure, but also to determine its thickness with a precision comparable to atomic force microscopy, which compares well with previous works^14^. Lastly, our work highlights the advantage of polarized optical contrast spectroscopy for characterizing delicate 2D materials, where methods requiring rather intense optical excitation, such as polarized second harmonic generation or Raman spectroscopy, may lead to unwanted material degradation.

## Supplementary Information

Supplementary Information 1.

Supplementary Information 2.

## Data Availability

All data that support the findings of this study are included within the article (and any supplementary files).
