# Supplementary material for: Polarized optical contrast spectroscopy of in plane anisotropic van der Waals materials
Source: Sci Rep. 2025 May 2;15:15344. doi: 10.1038/s41598-025-96894-8 (PMC12048637; doi:10.1038/s41598-025-96894-8)
Supplement: Supplementary file 1 — Supplementary Information 1. [file 41598_2025_96894_MOESM1_ESM.pdf]

Supporting Information for "Polarized optical contrast spectroscopy of in plane anisotropic van der Waals materials"

Ernst Knöckl[1,2], Alexandre Bernard[1,2], Alexander Holleitner[1,2], and Christoph Kastl[1,2]
[1] *Walter Schottky Institute and Physics Department, Technical University of Munich, Garching 85748, Germany* and
[2] *Munich Center for Quantum Science and Technologie (MCQST), München 80799, Germany*

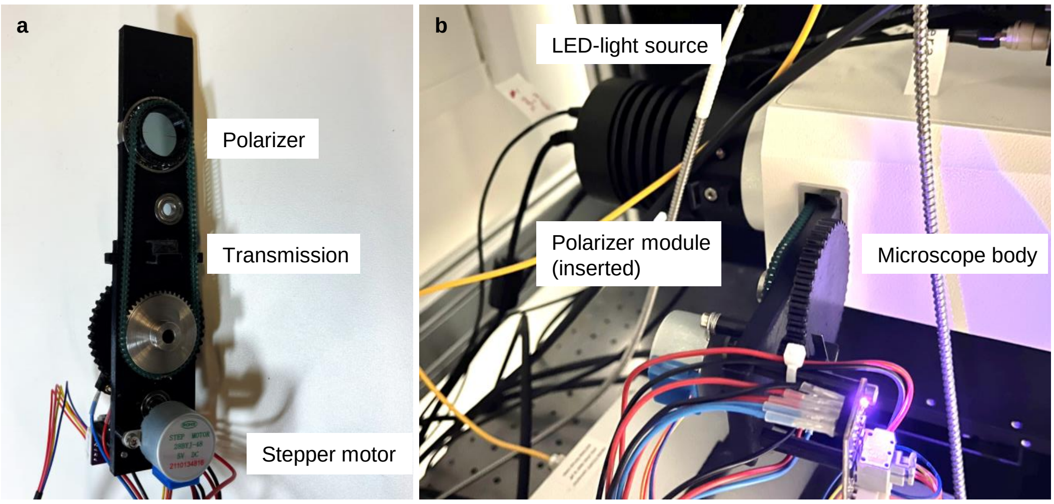

FIG. S1: **Custom-made, 3D-printed motorized polarization module.** **(a)** A stepper motor is controlled by a computer. Its rotation is first reduced by a larger gear and then transmitted through a belt drive onto a linear thin film polarizer mounted on a ball bearing. **(b)** The module is inserted into the illumination path of a commercial Raman microscope. The module is placed right after the LED white light source of the Raman microscope.

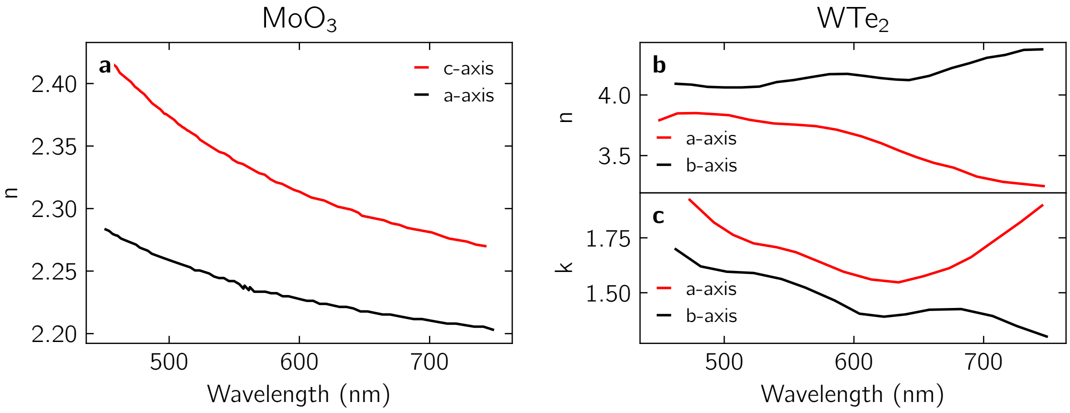

FIG. S2: **Refractive indices of MoO_3 and WTe_2.** **(a)** For MoO_3 , we consider only the refractive index n. The data is taken from Ref. [1] **(b)** For WTe_2 , we consider both refractive index n and absorption k. The data is taken from Ref. [2]. Both materials exhibit a significant birefringence whether the light is polarized parallel to one crystal axis or the other (red and black lines).

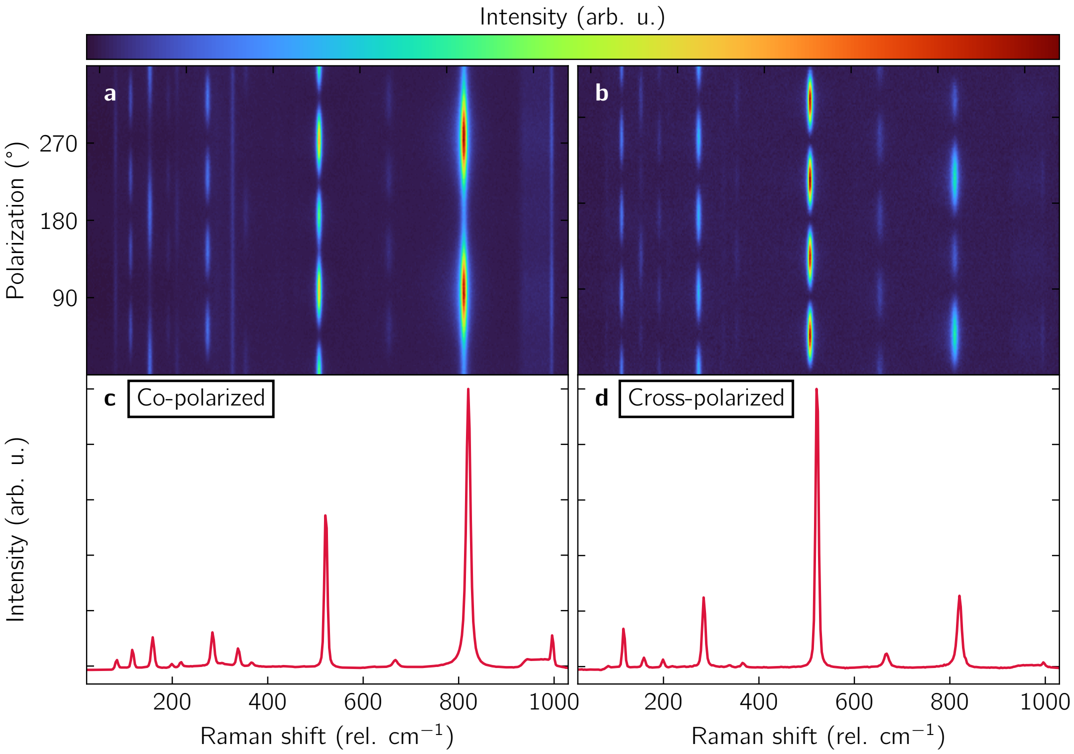

e

Symmetry	A_g^a	B_{2g}	A_g^c	B_{2g}	A_g^c	B_{2g}
Wavenumber (rel. cm^{-1})	82.2±0.4	115.3±0.1	157.5±0.1	197.7±0.3	216.7±0.3	283.0±0.1
Symmetry	A_g^c	A_g^c	B_{2g}	A_g^a	A_g^a	
Wavenumber (rel. cm^{-1})	336.6±0.1	364.9±0.5	665.6±0.1	818.8±0.1	994.7±0.4	

FIG. S3: **Polarized Raman spectroscopy of MoO_3.** The data was obtained on the 146 nm thin MoO_3 flake on Si/SiO_2 discussed in the main manuscript using a 532 nm excitation with $P = 5\,\mathrm{mW}$ in confocal configuration with a $\times 10$ objective with numerical aperture NA $= 0.25$, yielding a power density of $\sim 5.6\,\mathrm{mW µm^{-2}}$ and a beam spot of 1 µm. The Raman measurement was done at ambient conditions. **(a)** and **(b)** are Raman spectra as a function of polarization angle in co- and cross-polarized configurations, respectively. **(c)** and **(d)** are the corresponding spectra integrated over all polarization angles. **(e)** Raman peak positions reported for MoO_3. The peak positions are obtained by a standard least squares Lorentzian fitting routine from the data in **c**.

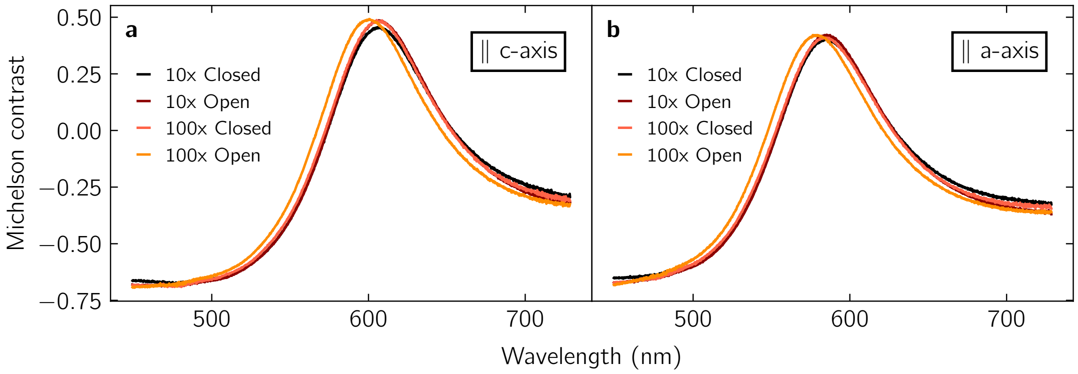

FIG. S4: **Influence of numerical aperture and aperture stop.** Polarized optical contrast of MoO_3 for different objective lenses (10× with NA = 0.25 and 100× with NA = 0.9) and configurations of the aperture stop. **(a)** Illumination polarized along the c-axis. **(b)** Illumination polarized along the a-axis. When the aperture stop is closed, the wide-field illumination is approximately parallel for all objectives, independent of their NA. Consequently, the contrast spectra do not change with the NA of the objective lens. When the aperture stop is open, the spectra strongly depend on the numerical aperture (yellow curve).

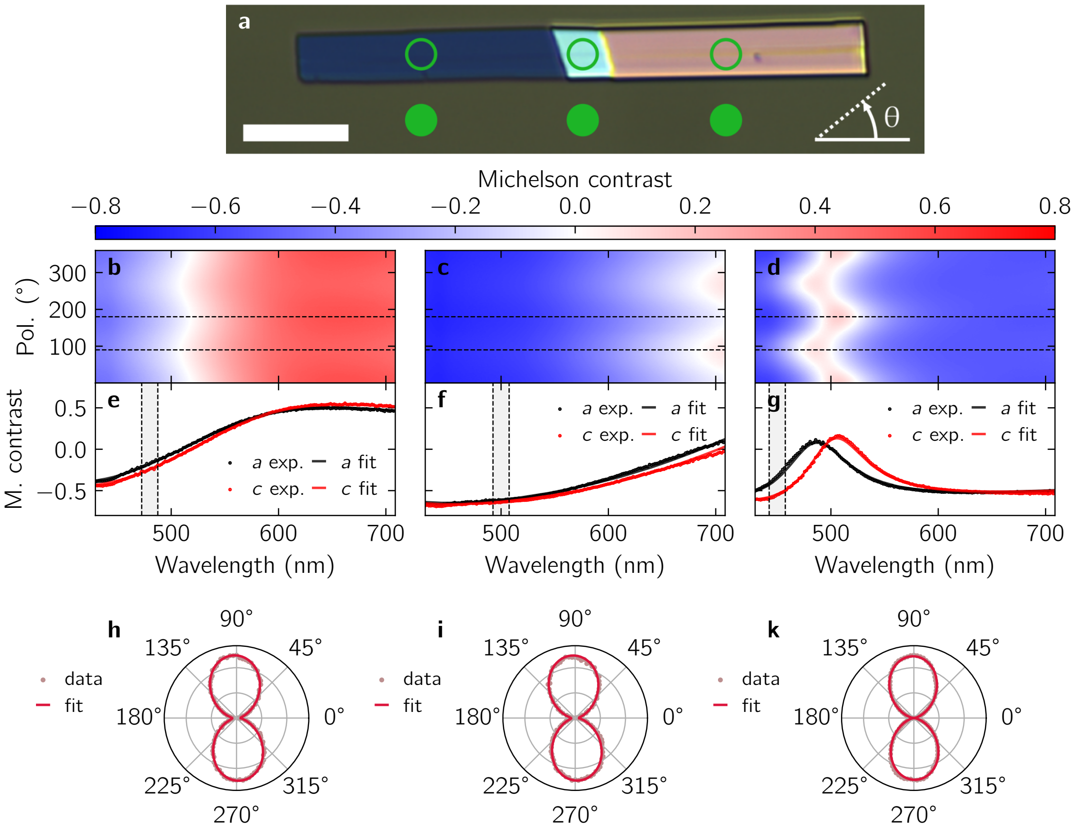

FIG. S5: **Polarized optical contrast spectroscopy on MoO_3 with different thicknesses.** **a** Optical image of a MoO_3 flake on a Si/SiO_2 substrate. The three different colors of the flake indicate regions with different thicknesses. Each empty disc marks the sample measurement spot (R_{on}) corresponding to the reference measurement spot indicated with the full disc underneath (R_{off}). The measured spectra allow us to define the Michelson contrast on the regions of different thickness. The polarization angle θ is defined as indicated. Scale bar, 10 µm. **b-d** Contrast spectra for various polarization orientations θ corresponding to the respective measurement area in **a**. **e-g** Michelson contrast spectra measured along the crystal's a-axis (black dots) and b-axis (red dots). The solid lines are fits with the transfer matrix model. The fits indicate that the dark blue region is 12 nm thick, the light blue one 40 nm and the red colored region stands out to 111 nm. **h-k** Normalized polar plot of the contrast integrated over the wavelength region represented by the grey area in **e-g**. The solid lines are sinusoidal fits. With a suitable choice of the integration window, the crystal axes determination via polarized optical contrast remains precise and consistent across different thicknesses. The orientation of the crystal's c-axis (i.e. the minimum of the fitted sinusoids) is determined to $(3 \pm 1)^\circ$ for the dark blue area, $(3 \pm 1)^\circ$ for the light blue area, and $(1 \pm 1)^\circ$ for the light red area, which matches reasonably well with the optical image as well as the 1° angular step size chosen for the measurements. Additionally, the fit determines precisely the film thickness.

Supporting References

[1] Daniel Andres-Penares, Mauro Brotons-Gisbert, Cristian Bonato, Juan F. Sánchez-Royo, and Brian D. Gerardot. Optical and dielectric properties of MoO_3 nanosheets for van der Waals heterostructures. *Applied Physics Letters*, 119(22):223104, 2021.

[2] Krastyo Buchkov, Rosen Todorov, Penka Terziyska, Marin Gospodinov, Velichka Strijkova, Dimitre Dimitrov, and Vera Marinova. Anisotropic optical response of WTe_2 single crystals studied by ellipsometric analysis. *Nanomaterials*, 11(9):2262, 2021.
